# Conversational LLM Chatbot ChatGPT-4 for Colonoscopy Boston Bowel Preparation Scoring: An Artificial Intelligence-to-Head Concordance Analysis

**DOI:** 10.3390/diagnostics14222537

**Published:** 2024-11-13

**Authors:** Raffaele Pellegrino, Alessandro Federico, Antonietta Gerarda Gravina

**Affiliations:** Hepatogastroenterology Division, Department of Precision Medicine, University of Campania Luigi Vanvitelli, Via L. de Crecchio, 80138 Naples, Italy

**Keywords:** ChatGPT, bowel preparation, colonoscopy, artificial intelligence

## Abstract

Background/objectives:To date, no studies have evaluated Chat Generative Pre-Trained Transformer (ChatGPT) as a large language model chatbot in optical applications for digestive endoscopy images. This study aimed to weigh the performance of ChatGPT-4 in assessing bowel preparation (BP) quality for colonoscopy. Methods: ChatGPT-4 analysed 663 anonymised endoscopic images, scoring each according to the Boston BP scale (BBPS). Expert physicians scored the same images subsequently. Results: ChatGPT-4 deemed 369 frames (62.9%) to be adequately prepared (i.e., BBPS > 1) compared to 524 frames (89.3%) assessed by human assessors. The agreement was slight (κ: 0.099, *p* = 0.0001). The raw human BBPS score was higher at 3 (2–3) than that of ChatGPT-4 at 2 (1–3), demonstrating moderate concordance (W: 0.554, *p* = 0.036). Conclusions: ChatGPT-4 demonstrates some potential in assessing BP on colonoscopy images, but further refinement is still needed.

## 1. Introduction

Large language model (LLM)-based conversational chatbots are artificial intelligence tools rapidly gaining traction in gastroenterology. Chat Generative Pre-Trained Transformer (ChatGPT) is a conversational chatbot developed by OpenAI based on LLM technology [1]. Its multiparametric functioning relies on reinforcement learning, allowing the chatbot, over time, to produce increasingly refined outputs as it receives more inputs [1]. ChatGPT (https://chatgpt.com/) has demonstrated the ability to produce text-based outputs capable of providing scientifically valid answers to frequently asked questions by patients regarding the management of inflammatory bowel diseases [2] and gastroenterological questions from specialist medical exams [3], as well as help in the formulation of research questions [4].

In digestive endoscopy, typical LLM chatbots (e.g., ChatGPT) could provide reliable medical information on frequently asked patient questions about colonoscopy preparation [5] and assist healthcare providers in decision-making regarding post-colonoscopy surveillance strategies [4]. A high-quality colonoscopy relies on adequately visualising the bowel walls to identify all mucosal and luminal qualitative–quantitative alterations. Proper preliminary bowel preparation (BP) ensures this visualisation [6]. Traditionally, scales assess BP based on the remaining opaque faecal content. Efforts have been made to automate this evaluation through artificial intelligence devices, aiming to provide faster, more reliable, precise, and real-time assessment of BP [7].

Apart from LLMs, various artificial intelligence applications have been used to assess bowel preparation quality for colonoscopy using text-based tools. Promising studies have employed natural language processing on written colonoscopy reports [8,9]. Nonetheless, convolutional neural networks are another technology that has shown equal potential in performing this task [10]. However, despite these advancements, the widespread availability and accessibility of advanced artificial intelligence systems for performing optical tasks in digestive endoscopy is not always satisfactory. Therefore, pursuing more easily disseminable artificial intelligence tools can parallel the development of higher-cost, less accessible systems.

To date, no study has evaluated widely accessible LLM chatbots performance, which are increasingly updated and capable of performing complex tasks in calculating the BP for colonoscopy. Notably, the latest versions of ChatGPT, such as ChatGPT-4mni(o), could be particularly well suited for evaluating BP in colonoscopy. These versions enable the integration of the chatbot’s conversational interface with the ability to upload many images [11]. This functionality could allow endoscopists to upload images associated with the colonoscopy examination immediately after completion.

## 2. Materials and Methods

### 2.1. Study Design

An LLM analysis was conducted using ChatGPT-4 (OpenAI, San Francisco, CA, USA) on fully anonymised endoscopic images randomly and retrospectively obtained through the Endobase system and captured using an EVIS X1 device (© Olympus 2024). Consequently, no photos can be traced back to the patients from whom they were obtained, nor are there any patient identification details in the study records.

**Figure 1:**
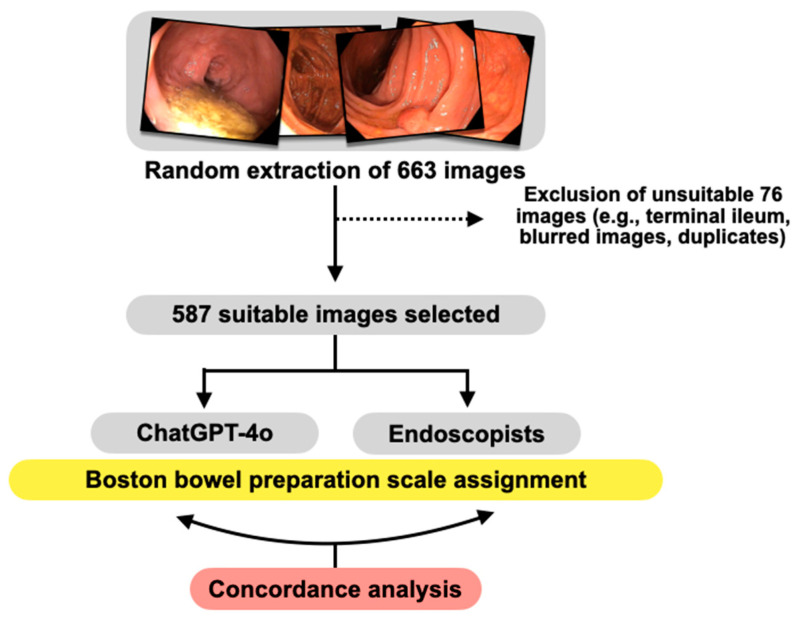
A summary of the study protocol.

The chatbot was asked to calculate each image’s Boston BP scale (BBPS) score [12], following specific inputs subsequently detailed. Three expert gastroenterology physicians also provided scores for the images evaluated by the chatbot, agreeing on a single score for each image. In case of disagreement, the absolute majority dictated the final score. Specific sub-characteristics were identified from the images, including colonic diverticula, caecal fundus (identified by the appendiceal orifice and/or the ileocaecal valve), polypoid and non-polypoid formations, narrow band imaging (NBI) use, blood, mucus, or images taken using retroflexion manoeuvres (performed in the caecum and rectum). Figure 1 summarises the study protocol.

### 2.2. Characteristics and Technical Specifications of the Images Included in the Analysis

As previously described, the images were extracted strictly anonymously from our endoscopy database at the digestive endoscopy unit of the Hepatogastroenterology Division, University of Campania Luigi Vanvitelli, in July 2024. Each image was saved in bitmap format (i.e., BMP) or portable network graphics format (i.e., PNG). The resolution of the images was 1920 × 1072 pixels, and the file size was generally 7.85 megabytes. No specific selection criteria were applied to the images, and no prior exclusions were made. Inappropriate images, such as those out of focus (blurred) or irrelevant (e.g., from the small intestine), were excluded post-analysis following evaluation by the physicians.

### 2.3. Images Processing by ChatGPT-4 and Human Evaluators

ChatGPT-4 allowed a maximum upload of images (i.e., ten images with our specified resolution and size) per analysis cycle. Therefore, sixty-six cycles of ten images each and one cycle of three images were conducted to complete the analysis. These cycles were carried out on different days due to the time-based limit on the number of files that could be uploaded within specific time periods. The outputs from the bot did not significantly differ across the various analysis cycles. The input provided to the chatbot once the image files were uploaded was as follows:

*Analyze these ten images taken from a colonoscopy and assign each one a score according to the BBPS scale, which should be either 0, 1, 2, or 3 based on the following criteria (Please assign the score yourself without asking me for input): 0 = Unprepared colon segment with mucosa not seen due to solid stool that cannot be cleared. 1 = Portion of mucosa of the colon segment seen, but other areas of the colon segment not well seen due to staining, residual stool and/or opaque liquid. 2 = Minor amount of residual staining, small fragments of stool and/or opaque liquid, but mucosa of colon segment seen well. 3 = Entire mucosa of colon segment seen well with no residual staining, small fragments of stool or opaque liquid. Subsequently, create a very simple and not interactive table with two columns, one with the file name and the other with the code you have calculated (0, 1, 2, or 3).*

The request for a simple table was necessary for us to easily copy and paste the data into the study’s database, as opposed to the interactive table ChatGPT-4 often provided as an output. ChatGPT always provided a single score per image without giving multiple or uncertain scores for each of our inputs. Each of these scores was then collected and entered into the study records. No image was subjected to more than one evaluation by ChatGPT-4. Some representative examples of outputs from ChatGPT-4 are provided in Table 1.

The three physicians evaluating the endoscopic images provided a BBPS score [12] based on the same input given to ChatGPT-4. The human evaluation preceded that of ChatGPT-4, ensuring that the endoscopists were unaware of the scoring performed by the chatbot in advance.

### 2.4. Study Outcomes

This analysis primarily aimed to assess the degree of agreement between the chatbot assessor and human assessor in evaluating BP. We calculated the instances where both assessors provided a compatible BBPS score for a given frame, considering BBPS > 1 to be adequate BP and BBPS < 2 to be suboptimal BP. Additionally, we compared the raw BBPS scores for each frame given by both assessors as a secondary outcome.

### 2.5. Statistical Analysis

Regarding the statistical evaluations, continuous variables were expressed as medians (interquartile range), and non-continuous variables were expressed as frequencies and percentages of the total. The concordance analysis (for categorical variable) used Cohen’s κ [13], expressing the κ concordance coefficient and the corresponding 95% confidence interval (CI). The interpretation of the concordance index followed this logic: no agreement for κ < 0, slight agreement for 0 ≤ κ ≤ 0.2, fair agreement for 0.21 ≤ κ ≤ 0.4, moderate agreement for 0.41 ≤ κ ≤ 0.6, substantial agreement for 0.61 ≤ κ ≤ 0.8, and almost perfect agreement for κ > 0.8. The difference between categorical concordance rates was analysed using the χ^2^ test. Kendall’s concordance coefficient W was employed for the concordance analysis of the raw BBPS scores (considered as continuous variables). The interpretation of W was as follows: 0.00 ≤ W < 0.20—weak concordance; 0.20 ≤ W < 0.40—slight concordance; 0.40 ≤ W < 0.60—moderate concordance; 0.60 ≤ W < 0.80—substantial concordance; W ≥ 0.80—almost perfect concordance [14]. For this final analysis, concordance was also assessed using Bland–Altman plots [15], expressing the level of bias with the corresponding standard deviation and 95% limits of agreement. A two-tailed *p*-value of less than 0.05 was considered significant.

Statistical analysis was performed using IBM^®^ SPSS^®^ software (version 25, IBM Corp.©, Armonk, NY, USA), and graphs were generated using GraphPad PRISM^®^ software (version 9.5.0, GraphPad Software LLC©, Boston, MA, USA). The sample size was calculated using G*Power software (version 3.1.9.6, Faul, Erdfelder, Lang, & Buchner, Dusseldorf, Germany).

## 3. Results

The total pool of endoscopic images initially comprised 663 images. However, 76 images were subsequently excluded for the following reasons: 6% originated from the terminal ileum, 3% were blurred, 1.8% were exact duplicates of the same image, and 0.1% came from an operated colon, which did not allow for the application of the BBPS. Consequently, 587 images were processed (Figure 1).

The number of frames deemed adequate BP was 369 (62.9%) and 524 (89.3%) for the chatbot and human assessors, respectively. Conversely, the suboptimal BP frames were 218 (37.1%) and 63 (10.7%). The degree of agreement was slight (κ: 0.099, *p* = 0.0001; χ^2^ = 10.25, *p* = 0.001).

In the raw evaluation of BBPS scores, the median scores of the human assessor were higher at 3 (2–3) compared to ChatGPT-4 at 2 (1–3), with moderate agreement in the concordance analysis (W: 0.554, *p* = 0.036).

**Figure 2:**
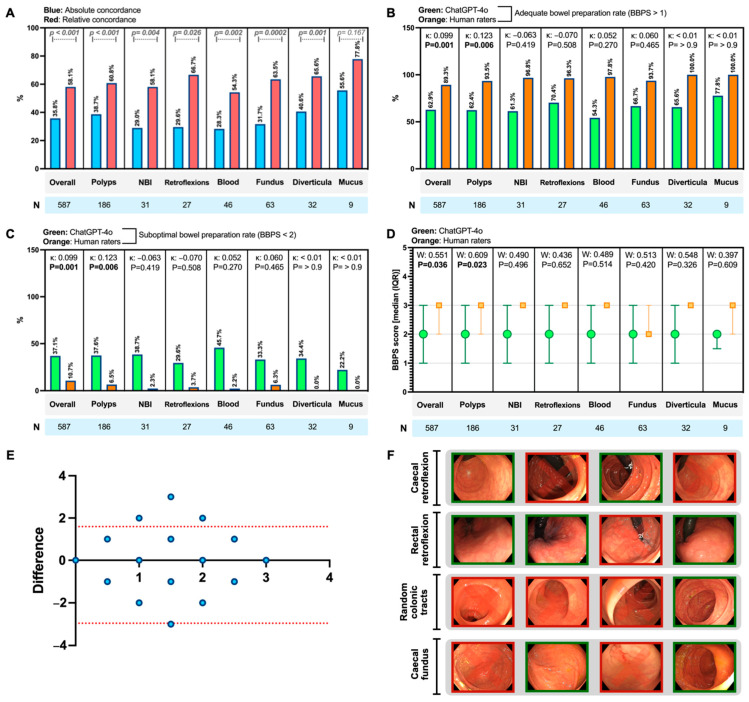
(**A**) Absolute concordance rates, in which both the chatbot and human evaluator provided the same Boston Bowel Preparation Scale (BBPS) scores for the same images (in blue), and relative concordance rates, in which both provided the same assessment of bowel preparation adequacy (BBPS > 1) for the same images (in red). These rates concern the overall set of images and specific frames showing particular findings [such as polyps, diverticula, narrow band imaging (NBI), retroflexion manoeuvres, etc.]. Differences in rates are calculated using the chi-squared test, with the corresponding *p*-value and sample size (N) indicated. (**B**,**C**) A comparison of the rates of adequate bowel preparation (i.e., BBPS > 1) and suboptimal bowel preparation (i.e., BBPS < 2) between Chat Generative Pre-Trained Transformer 4 (ChatGPT-4) and human raters, including their respective concordance coefficients (κ) and *p*-values (significant values are highlighted in bold). (**D**) A comparison of the medians (interquartile range, IQR) of raw BBPS scores provided by both raters (i.e., ChatGPT-4 and the endoscopists), along with their respective concordance coefficients (W) and *p*-values (significant values are highlighted in bold). (**E**) Difference-based Bland–Altman plot on the concordance between the BBPS scores of the chatbot and the human raters (blue dots), with a recorded bias of −0.6814 ± 1.161 (95% limits of agreement, represented by red dotted lines, ranging from −2.956 to 1.593). (**F**) Representative endoscopic images showing how, for similar images, the chatbot scores differently in contexts varying in terms of localisation and endoscopic manoeuvre (images considered to have suboptimal preparation are marked in red with a cross, while the others are deemed to have adequate preparation).

The analysis of the subcategories of the endoscopic images (e.g., polyps, use of NBI, retroflexions) showed that concordance appears to be higher when polyps are present (κ: 0.123, *p* = 0.0006; W: 0.551, *p* = 0.023) compared to other subcategories (*p* > 0.05). Figure 2A–D summarise the concordance analyses performed.

## 4. Discussion

For the first time, these data indicate that a conversational chatbot may have significant potential for systematically evaluating the quality of BP across large volumes of frames obtained from colonoscopies (output examples in Table 1). Notwithstanding this, there is a risk of artificial hallucinations [16] that must be addressed, as well as a pressing need to evaluate this performance in randomised controlled settings. Figure 2F highlights similar frames in which ChatGPT-4 provides completely different, and even hallucinated [16], evaluations for BP.

In addition to this drawback, the highly evolutionary nature of such a chatbot, based on constant reinforcement training, makes LLM-related evidence particularly susceptible to engineering changes in the system. On the other hand, it also indicates these chatbots’ strongly evolutionary (and potentially improving) nature. This highlights the need for future follow-up studies to evaluate the evolution of performance and concordance indices. Additionally, assessing larger volumes of frames will help to identify challenges for the chatbot (in terms of specific image characteristics) and determine more effective inputs to digit.

This study represents a shift in the use of LLMs in digestive diseases, as previous applications were primarily conversational, such as answering common patient questions, serving as supplementary study tools for medical exams, and aiding in clinical decision-making [2,4]. These data, moreover, highlight human assessors’ need to double-check chatbots before using them in clinical practice to ensure clinical–scientific accuracy and medico-legal compliance [17]. This is particularly important given the risk of artificial hallucinations described for LLMs like ChatGPT [18], which, as mentioned, were also highlighted in some instances in our study.

The use of LLMs for interpreting image-based rather than text-based inputs is still highly experimental in the medical field. These tools are primarily designed for conversational functions [1,4,19]. However, they are evolving steadily, and newer versions of ChatGPT now allow for image uploads, unlike earlier versions, opening up new research possibilities that were previously inaccessible.

Among the few studies already performed, Arruzza et al. [20] conducted a preliminary study similar to ours to evaluate knee, elbow, ankle, hand, shoulder, and pelvic projection radiographs. Their study recorded an exact match accuracy of 20%, thus yielding a lower performance than our work’s results. This likely suggests that ChatGPT’s performance is variable and depends on the type of images that it is given as an input. In contrast, Elek et al. [21] assessed the interpretative performance of Microsoft Bing’s LLM on eighty images from computed tomography (CT) and magnetic resonance imaging (MRI), recording an accuracy of approximately 95.4% for recognising CT images and 86.1% for MRI images. However, these studies are challenging to compare with ours, as our tasks likely involved a higher level of complexity, such as recognising both the quantitative and qualitative characteristics of residual faecal matter, interpreting them, and identifying the level of BP according to the BBPS scoring scale.

An additional study in the field of nephrology demonstrated a better performance by ChatGPT (versions 3.5 and 4) and Bard in responding to non-image-based nephrology-specific questions compared to those requiring image interpretation [22]. This further highlights the need to refine these LLMs for image-based tasks [23].

Moreover, our data suggest that specific endoscopic findings, such as polyps, may significantly increase the level of concordance between ChatGPT and the endoscopists’ evaluations. This is a noteworthy finding as it may indicate that ChatGPT could perform better when polyps are present in the assessment of BP. Therefore, it might be valuable to design future studies to evaluate its capabilities in optical parameters such as the polyp detection rate or adenoma detection rate or, more generally, to assess its ability to identify the pit pattern of polypoid and non-polypoid formations in the colon.

This analysis presents several limitations, including an uncontrolled evaluation. Nonetheless, the image assessment was random, so the chatbot evaluated images potentially sourced from different patients’ colons within the same cycles to anonymise the process. Conversely, assessing the chatbot’s performance on individual examinations (and on subsets of images derived from the same patient at one time) might be helpful. This would allow for a much more real-life analysis and enable the calculation of the total BBPS (viewed as the partial BBPS for the right, transverse, and left colon [12]).

Additionally, it would be beneficial in future studies to assess the performances of other LLMs under the same conditions besides ChatGPT. It would also be valuable to conduct repeated studies to evaluate the intra-rater consistency of the LLM model at similar time intervals and, therefore, comparable levels of model updates.

Despite these limitations, the large sample sizes of the frames and the effectively blind evaluation by human assessors (who did not know in advance the scores given by ChatGPT-4, which were collected afterwards) are strengths, at least in highlighting medium-to-large effect sizes.

In conclusion, these data suggest that ChatGPT-4 could be a promising tool for identifying optical parameters in digestive endoscopy (precisely the degree of BP), especially when precise and subtle frame-by-frame interpretations are not required. Further steps are necessary for its progressive implementation.

## Figures and Tables

**Table 1:** Representative examples of outputs provided by Chat Generative Pre-Trained Transformer 4-omni (ChatGPT-4o) in response to inputs for calculating the Boston Bowel Preparation Scale (BBPS) for individual colonoscopy endoscopic images uploaded during each usage cycle of ChatGPT. The table displays only the outputs, as the inputs have already been explained and detailed in the methods section.

Images Code	ChatGPT-4o Outputs ^1^
Images 61–67	Based on the provided BBPS (Boston Bowel Preparation Scale) criteria, I’ve analyzed the images and assigned the scores as follows: Image61.png 1; Image62.png 1; Image63.png 3; Image64.png 3; Image65.png 2; Image66.png 2; Image67.png 3; Image68.png 3; Image69.png 1; Image70.png 1. Here is the table with the file names and their corresponding BBPS scores. If you have any further analysis or additional instructions, please let me know!
Images 161–170	Here are the BBPS scores for the additional images provided: Image 161: Minor amount of residual staining, small fragments of stool and/or opaque liquid, but mucosa of colon segment seen well. Score: 2; Image 162: Entire mucosa of colon segment seen well with no residual staining, small fragments of stool or opaque liquid. Score: 3; Image 163: Entire mucosa of colon segment seen well with no residual staining, small fragments of stool or opaque liquid. Score: 3; Image 164: Entire mucosa of colon segment seen well with no residual staining, small fragments of stool or opaque liquid. Score: 3; Image 165: Entire mucosa of colon segment seen well with no residual staining, small fragments of stool or opaque liquid. Score: 3; Image 166: Minor amount of residual staining, small fragments of stool and/or opaque liquid, but mucosa of colon segment seen well. Score: 2; Image 167: Minor amount of residual staining, small fragments of stool and/or opaque liquid, but mucosa of colon segment seen well. Score: 2; Image 168: Portion of mucosa of the colon segment seen, but other areas of the colon segment not well seen due to staining, residual stool and/or opaque liquid. Score: 1; Image 169: Portion of mucosa of the colon segment seen, but other areas of the colon segment not well seen due to staining, residual stool and/or opaque liquid. Score: 1; Image 170: Portion of mucosa of the colon segment seen, but other areas of the colon segment not well seen due to staining, residual stool and/or opaque liquid. Score: 1 Here is the table with the file names and their corresponding BBPS scores: Image161.png 2; Image162.png 3; Image163.png 3; Image164.png 3; Image165.png 3; Image166.png 2; Image167.png 2; Image168.png 1; Image169.png 1; Image170.png 1.
Images 261–270	Based on the Boston Bowel Preparation Scale (BBPS), here are the scores assigned to each new image: Image261.BMP: Score = 0 (Unprepared colon segment with mucosa not seen due to solid stool that cannot be cleared); Image262.BMP: Score = 0 (Unprepared colon segment with mucosa not seen due to solid stool that cannot be cleared); Image263.BMP: Score = 0 (Unprepared colon segment with mucosa not seen due to solid stool that cannot be cleared); Image264.BMP: Score = 0 (Unprepared colon segment with mucosa not seen due to solid stool that cannot be cleared); Image265.BMP: Score = 2 (Minor amount of residual staining, small fragments of stool and/or opaque liquid, but mucosa of colon segment seen well); Image266.BMP: Score = 1 (Portion of mucosa of the colon segment seen, but other areas of the colon segment not well seen due to staining, residual stool and/or opaque liquid); Image267.BMP: Score = 3 (Entire mucosa of colon segment seen well with no residual staining, small fragments of stool or opaque liquid); Image268.BMP: Score = 3 (Entire mucosa of colon segment seen well with no residual staining, small fragments of stool or opaque liquid); Image269.BMP: Score = 3 (Entire mucosa of colon segment seen well with no residual staining, small fragments of stool or opaque liquid); Image270.BMP: Score = 3 (Entire mucosa of colon segment seen well with no residual staining, small fragments of stool or opaque liquid). Here’s the table with the file names and the assigned scores: Image261.BMP 0; Image262.BMP 0; Image263.BMP 0; Image264.BMP 0; Image265.BMP 2; Image266.BMP 1; Image267.BMP 3; Image268.BMP 3; Image269.BMP 3; Image270.BMP 3. If you need further assistance or have any specific requirements, please let me know!

^1^ ChatGPT provided the list of image outputs as lists or tables. It was reformatted into a single paragraph to enhance the readability of this article. Acronyms: BMP: bitmap format; PNG: portable network graphics format.

## Data Availability

The original contributions presented in this study are included in the article; further inquiries can be directed to the corresponding author.
